# Protective effects of yeast extract against alcohol-induced liver injury in rats

**DOI:** 10.3389/fmicb.2023.1217449

**Published:** 2023-07-20

**Authors:** Zihan Lin, Yongjun Li, Man Wang, Huan Li, Yihong Wang, Xin Li, Ying Zhang, Di Gong, Lin Fu, Siying Wang, Danfeng Long

**Affiliations:** ^1^School of Public Health, Lanzhou University, Lanzhou, China; ^2^Gansu Provincial Center for Disease Control and Prevention, Lanzhou, China

**Keywords:** yeast extract, alcoholic liver injury, gut microbiota, metabolomics, rat

## Abstract

Oxidative stress, inflammatory response, and gut-liver axis dysbiosis have been suggested as the primarily involved in the pathogenesis of alcoholic liver injury. Previous research established that yeast extract (YE) has antioxidant, immune-boosting or microbiota-regulating properties. However, there is currently lack of information regarding the efficacy of YE on alcoholic liver injury. This study seeks to obtain data that will help to address this research gap using a Wistar male rat experimental model. Histologic and biochemical analysis results showed that the groups treated with both low-dose yeast extract (YEL) and high-dose yeast extract (YEH) had lower degrees of alcohol-induced liver injury. The abundance of *Peptococcus* and *Ruminococcus* reduced in the low-dose yeast extract (YEL) group, while that of *Peptococcus*, *Romboutsia, Parasutterella*, and *Faecalibaculum* reduced in the high-dose (YEH) group. Furthermore, Spearman analysis showed that the gut microbes were significantly associated with several liver-related indicators. For the analysis of differential metabolites and enriched pathways in the YEL group, the abundance of lysophosphatidylcholine (16:0/0:0) significantly increased, and then the levels of histamine, adenosine and 5′ -adenine nucleotide were remarkedly elevated in the YEH group. These findings suggest that both high and low doses of YE can have different protective effects on liver injury in alcoholic liver disease (ALD) rats, in addition to improving gut microbiota disorder. Besides, high-dose YE has been found to be more effective than low-dose YE in metabolic regulation, as well as in dealing with oxidative stress and inflammatory responses.

## 1. Introduction

Alcoholic liver disease (ALD) is a widespread worldwide condition and is one of the main causes of chronic liver damage (Singal et al., 2018). According to a systematic analysis of the Global Burden of Disease Study that was published by the World Health Organization (WHO), alcohol abuse was responsible for an estimated three million deaths in 2016, thereby accounting for nearly 6% of total global deaths (GBD 2016 Alcohol Collaborators, 2018). Alcoholic fatty liver (AFL), alcoholic hepatitis (AH), alcoholic cirrhosis (AHF), and alcoholic hepatocellular carcinoma (AHC) are the main pathological changes of alcoholic liver disease, and they can exist alone, partially overlap, or even coexist (Magdaleno et al., 2017). Alcohol abuse can cause damage to multiple organs, including the intestines, brain, and the liver, which is mainly involved in alcohol metabolism. In China, alcohol has become the second leading cause of liver injury, after hepatitis virus (Crabb et al., 2020). Mild liver damage caused by alcohol can be alleviated by long-term alcohol abstinence. However, when the situation develops to a severe stage in the later stage, the damage to the liver is extreme, irreversible, and even life-threatening (Joshi et al., 2016). Therefore, early intervention against and protection from ALD is of paramount importance.

Ethanol is mainly metabolized in the liver to form acetaldehyde, a reaction that is catalyzed by alcohol dehydrogenase (Setshedi et al., 2010). The acetaldehyde is then converted to acetic acid by acetaldehyde dehydrogenase (Kong et al., 2019). When excessive alcohol is consumed, acetaldehyde accumulates in the liver, and this, in turn, activates cytochrome P450 2E1 (CYP2E1) (Hyun et al., 2021). The activated CYP2E1 promotes acetaldehyde production through the formation of reactive oxygen species (ROS) (Cederbaum et al., 2009; Leung and Nieto, 2013). Meanwhile, the cumulative acetaldehyde reacts with antioxidants such as glutathione, reduces the level of antioxidants in the body, leads to an imbalance between oxidation and antioxidants in the body, and aggravates liver damage (Peixoto et al., 2004). This imbalance will increase neutrophil inflammation and protease secretion to generate ROS, eventually leading to oxidative stress (Lee et al., 2017; Albillos et al., 2020). The ROS also impair intestinal integrity (Seok et al., 2013). Additionally, research has found that alcohol consumption can increase the expression of miR-212, which down-regulates the expression of Zonula occludens 1 (ZO-1), ultimately leading to increased intestinal permeability (Tang et al., 2008). Therefore, lipopolysaccharide (LPS) produced by intestinal Gram-negative bacteria can be transported into the liver through the portal vein, thereby triggering the secretion of Kupffer cells such as interleukin-6 (IL-6), leukocytes-1β (IL-1β) (Aldred and Nagy, 1999; Wang et al., 2012), and tumor necrosis factor-α (TNF-α) (Wei et al., 2022). All these factors contribute to the development of ALD.

Yeast extract (YE) is a valuable source of amino acids, proteins, vitamin B-complex, peptides, and free nucleotides (Chae et al., 2001; Takalloo et al., 2020). It has been widely used as a nutritional resource, food flavoring, additive, and vitamin supplement (Eom et al., 2021). By adding YE to the feed, it can significantly improve the growth performance and antioxidant capacity of juvenile pacific white shrimp, along with the relative abundance of beneficial bacteria in the gut microbiota, while reducing the relative numbers of the conditional pathogenic bacteria (Zheng et al., 2021). A daily supplement of 100 mg/kg YE has been shown to reduce blood triglycerides and total cholesterol in rats (Waszkiewicz-Robak, 2013; Tao et al., 2023). YE also exerted anti-inflammatory properties by down-regulating the gene expression of inflammatory factors such as TNF-α and IL-1β in the body (Yuan et al., 2017; Jin et al., 2018). In terms of disease prevention, research has shown that yeast cell wall extract has a certain alleviating effect on necrotic enteritis in broiler chickens (M'Sadeq et al., 2015). Therefore, the YE has great potential for disease prevention and control across various fields. However, to date, there is a lack of research reports on the application of YE in the study of alcoholic liver injury. In this study, a rat model was established to explore the protective effects of different doses of the YE on alcoholic liver injury. By combining 16S rRNA sequencing and TM widely targeted metabolomics analysis, the “gut-liver” axis mechanism of the YE’s protective action on the alcoholic liver injury was investigated.

## 2. Materials and methods

### 2.1. Materials

YE (MF-4, purity≥98%, nutrient composition are shown in Supplementary Table S1)was supplied from Mufan Biotechnology Co., Ltd. (Henan, China). Silymarin (H20181067, 140 mg/capsule) was obtained from Plantextrakt GmbH & Co. KG. The liquid diet was purchased from Trophic Animal Feed High-Tech Co., Ltd. (Nantong, China). Detection kits for alanine aminotransferase (ALT), aspartate aminotransferase (AST), total antioxidant capacity (T-AOC), glutathione peroxidase (GPX), malondialdehyde (MDA), superoxide dismutase (SOD), lactic dehydrogenase (LDH), and superoxide Anion (O2-) were purchased from Beijing Boxbio Science & Technology Co., Ltd. Detection kits for alkaline phosphatase (AKP), triglyceride (TG), total cholesterol (T-CHO), lipid peroxidation (LPO), alcohol dehydrogenase (ADH), Hydroxyl Radical Scavenging Ability (OH Scavenging Ability), total nitric oxide synthase (TNOS), and inducible nitric oxide synthase (iNOS) were purchased from Jiancheng Corp (Nanjing, China). ELISA kits for leukocytes-1β (IL-1β), tumor necrosis factor-α (TNF-α), interferon-γ (INF-γ) were supplied from Elabscience Biotechnology Co., Ltd. Fish oil was purchased from Shanghai Yien Chemical Technology Co., Ltd. (Shanghai, China). Leukocytes-1β (IL-1β), tumor necrosis factor-α (TNF-α) and interferon-γ (INF-γ) were supplied from Elabscience Biotechnology Co., Ltd. Fish oil was purchased from Shanghai Yien Chemical Technology Co., Ltd. (Shanghai, China).

### 2.2. Animals and experimental design

Four-week-old male Wistar rats were purchased from Lanzhou University Animal Experimental Center (Lanzhou, China). The rats were housed in a clean environment with a 12-h light–dark cycle at 20–22°C and 45 ± 5% humidity. All the rats were free to access food and water *ad libitum*. All experiments involving animals were approved by the Ethics Committee of School of Public Health, Lanzhou University (IRB22030601). After one-week of acclimatization, the rats were randomly divided into the following five groups (11 rats per group): liquid diet control group (LC), alcohol liquid diet group (AL), low-dosage yeast extract intervention group (YEL, 60 mg/kg/day), high-dosage yeast extract intervention group (YEH, 120 mg/kg/day), and silymarin intervention group (PC, 120 mg/kg/day). AL, PC, YEL, and YEH groups were all given liquid diet with an alcohol concentration of 8% (v/v). Both types of liquid diet (with and without alcohol) had calorie densities of 1 kcaL/mL. The source of fatty acids in the diet was fish oil. During the first 3 days of adaptive feeding, all groups were given a control liquid diet. From the fourth to the nineth day, all groups (except for the LC group) entered the adaptive feeding stage of an alcohol liquid diet. The ratio of liquid control feed to liquid alcohol feed was 2:1, 1:1, and 1:2, and each ratio was fed for 2 days. After the adaptive feeding period, the rats were provided with a complete liquid alcohol diet. At the start of the alcohol feeding procedure, YE and silymarin were well-dissolved in ultrapure water prior to daily administration to the animals via gavage, over a period of 6 weeks. Food intake and body weight were monitored daily and weekly, respectively. At the end of the experiments, the rats were gavaged with 40% alcohol (15 mL/kg). After 12 h, the rats were anesthetized with an intraperitoneal injection of 3% sodium pentobarbital and then sacrificed. The liver of each rat was weighed to calculate the liver organ coefficient (wet liver weight, mg/ body weight, g). The livers were removed and rinsed with cold PBS. Portions of the liver were used for histological evaluation, and others were stored at −80°C for further analysis. Fecal samples were immediately frozen in liquid nitrogen and stored at −80°C until analysis was done.

### 2.3. Hepatic histological analysis

Samples of the right liver lobe were fixed in 4% paraformaldehyde for 24 h, embedded in paraffin wax, and then cut into 5 μm-thick sections. The sections were blurred by Hematoxylin–eosin (H&E) staining and Masson’s trichrome. The pathological changes on the liver were observed using the BX53 system microscope with an attached camera (Olympus, Japan) at 400× magnification.

### 2.4. Analysis of liver function index

The liver samples (about 100–150 mg) were mixed with 0.9% normal saline at a ratio of 1:9 to make homogenates. Centrifugation was then done at 3000 rpm for 15 min, at 4°C. The supernatant was collected for subsequent analyzes. The levels of ALT, AST, AKP, ADH, and LDH were assessed according to the instructions on the commercial assay kits.

### 2.5. Detection of oxidative stress indicators

To evaluate the oxidative stress indicators, homogenates were prepared using liver samples weighing 200 mg combined with normal saline, at a ratio of 1:9. The levels of MDA, T-AOC, LPO, SOD activity, and GPX activity were detected, following the manufacturer’s instructions.

### 2.6. Inflammatory parameters analysis

The liver tissues (about 50–100 mg) were mixed with normal saline at a ratio of 1:9 to prepare homogenates. Supernatants were collected after centrifugation. The levels of TNOS and iNOS in the liver tissues were analyzed by assay kits (Nanjing, China), according to the manufacturers’ instructions. The levels of IL-1β, TNF-α, and INF-γ in liver tissue were measured using ELISA kits (Elabscience Biotechnology, China) followed by the method described in the instructions.

### 2.7. 16S rRNA gene sequencing analysis

The analysis was conducted at Metware Biotechnology Co., Ltd. (Wuhan, China). Genomic DNA from the fecal samples (six samples were randomly selected from each group) was extracted by the Magnetic Soil and Stool DNA kit (TianGen Biotech Co., Ltd., Beijing, China). The bacterial sample was used to amplify the V4 hypervariable region of the 16S rRNA gene with primers 515F(GTGCCAGCMGCCGCGGTAA) and 806R(GGACTACHVGGGTWTCTAAT) (Itoh et al., 2014). Purified PCR products were quantified, using enzyme-labeled quantification, and the sequencing library preparation was performed based on the manufacturer’s instructions (Illumina, San Diego, CA, United States). Quantification was performed by Qubit and Q-PCR;sequencing was done using NovaSeq6000 (Illumina, San Diego, CA, United States). For these data, the reads of each sample were assembled using FLASH (v1.2.11, http://ccb.jhu.edu/software/FLASH/) after truncating the barcode and primer sequences, resulting in the Raw Tags. The Raw Tags were then truncated at the first low-quality base site, with consecutive low-quality values (default quality threshold: ≤ 19) and a base number reaching the set length (default length value is 3). Subsequently, the truncated Tags were filtered to remove those with a continuous high-quality length below 75% of the Tags length. The effective Tags were sequenced using Novaseq 6,000 (Illumina, San Diego, CA, United States), and the Uparse algorithm (v7.0.1001, http://www.drive5.com/uparse/) was used to cluster all sequences into OTUs (Operational Taxonomic Units). For the species annotation analysis of OTUs sequences, the Mothur method and the SSUrRNA database of SILVA138.1^1^ were used, setting the threshold between 0.8 ~ 1.

Furthermore, the Qiime software (version 1.9.1) was used to the “observed number,” as well as the Shannon and Simpson indices. R software (version 4.1.2) was employed in creating the Rank abundance curve. The nonparametric test of the difference between groups in Alpha diversity index was done using the Kruskal-Wallis test. LEfSe analysis was performed using the LEfSe software (version 1.0, https://huttenhower.sph.harvard.edu/lefse/), and the default LDA score filtering value was set to 4. The Mothur software (University of Michigan, Michigan, United States) was used in Metastats analysis at each classification level to obtain the *p* value (*p* < 0.05). The Benjamini and Hochberg False Discovery Rate method was then used to correct the *p* value to obtain the q value.

### 2.8. Metabolomics analysis

In this research, the metabolites were analyzed at Metware Biotechnology Co., Ltd. (Wuhan, China). Fecal samples (six samples were randomly selected from each group) stored at −80°C refrigerator were thawed. A 400 μL solution (methanol: water = 7:3, v/v) containing an internal standard was added into each sample (20 mg) and vortexed for 3 min. All samples were sonicated in an ice bath for 10 min and vortexed for 1 min before being placed in −20°C for 30 min. These samples were then centrifuged at 12000 rpm for 10 min at 4°C. Each supernatant was recentrifuged at 12000 rpm for 3 min at 4°C. 200 μL aliquots of each supernatant were transferred for ultra-performance liquid chromatography tandem mass spectrometry (UPLC-MS/MS, Sciex, United States) analysis, which included both non-targeted and broad-spectrum targeted detection. The non-targeted metabolites were separated on a pre-column (ACQUITY HSS T3 C18,1.8 μm, 2.1 mm*100 mm), with the column temperature being set at 40°*C. mobile* phase A consisted of water with 0.1% formic acid, and mobile phase B was comprised of acetonitrile with 0.1% formic acid. The gradient program was as follows: 95:5 v/v at 0 min; 10:90 v/v at 10.0 and 11.0 min; 95:5 v/v at 11.1 min; 95:5 v/v at 14.0 min. The flow rate was 0.4 mL/min, with an injection volume of 5 μL. The Quadrupole-Time of Flight mass spectrometry analysis was performed with an electrospray ionization (ESI) temperature of 500°C, voltage of 5,500 V (positive) and − 4,500 V (negative), ion source gas I (GS I) at 50 psi, gas II (GS II) at 50 psi, and Curtain gas (CUR) at 25 psi. The delustering potential collision energy was set at 80 psi, and the collision energy spread was at 15 psi. For Broad-spectrum targeted detection, a waters ACQUITY UPLC HSS T3 C18 chromatographic column (1.8 μm, 2.1 mm*100 mm) was employed, with an injection volume of 2 μL. Tandem mass spectrometry (QTRAP^®^) analysis was carried out using the same ESI temperature, voltage, ion source gas, and curtain gas condition as those in the Quadrupole-Time of Flight mass spectrometry.

All sample extracts were mixed in equal amounts to form a QC sample. Based on the database MWDB (including secondary spectrum, retention time RT), DB-all public database (including HMDB, KEGG and other databases), AI prediction library, and MetDNA, metabolites identification and extraction of multiple ion pairs, as well as retention time RT information were performed The MRM accurate quantification of the metabolites in the new library was carried out using the Q-Trap instrument platform.

The OPLS-DA function of the MetaboAnalyst R package in the R software was used for analysis. Based on the OPLS-DA results, the Variable Importance in Projection (VIP) of the OPLS-DA model was determined from the obtained multivariate analysis, and the differential metabolites were further screened by combining with the fold change. The selected data with different amounts of the metabolites were treated with UV (unit variance scaling). The R software Complex Heatmap package was used to draw the cluster heat map. Pearson correlation analysis was used to analyze the association between different metabolites that were identified according to the screening criteria.

Finally, the KEGG (Kanehisa and Goto, 2000)^2^ and HMDB^3^ database were used to annotate metabolites and identify the pathways of differential metabolite enrichment.

### 2.9. Statistical analysis

All statistical analysis was carried out using the SPSS statistical software (version 22.0, IBM, White Plains, New York, United States), and the measurement data were expressed as mean ± standard error of mean deviation. One-way ANOVA was used for comparison among multiple groups and the significance of difference between the two groups was determined by LSD test. When the variances are not equal, Dunnett’s T3 was used to test the differences between the two groups. Values of *p* < 0.05 were considered significant. All images were generated by OriginPro (2019, OriginLab, Massachusetts, United States) and GraphPad Prism (version 9.5.1, Graphpad Software, California, United States).

## 3. Result

### 3.1. Effect of YE on alcohol-induced hepatoxicity

**Figure 1:**
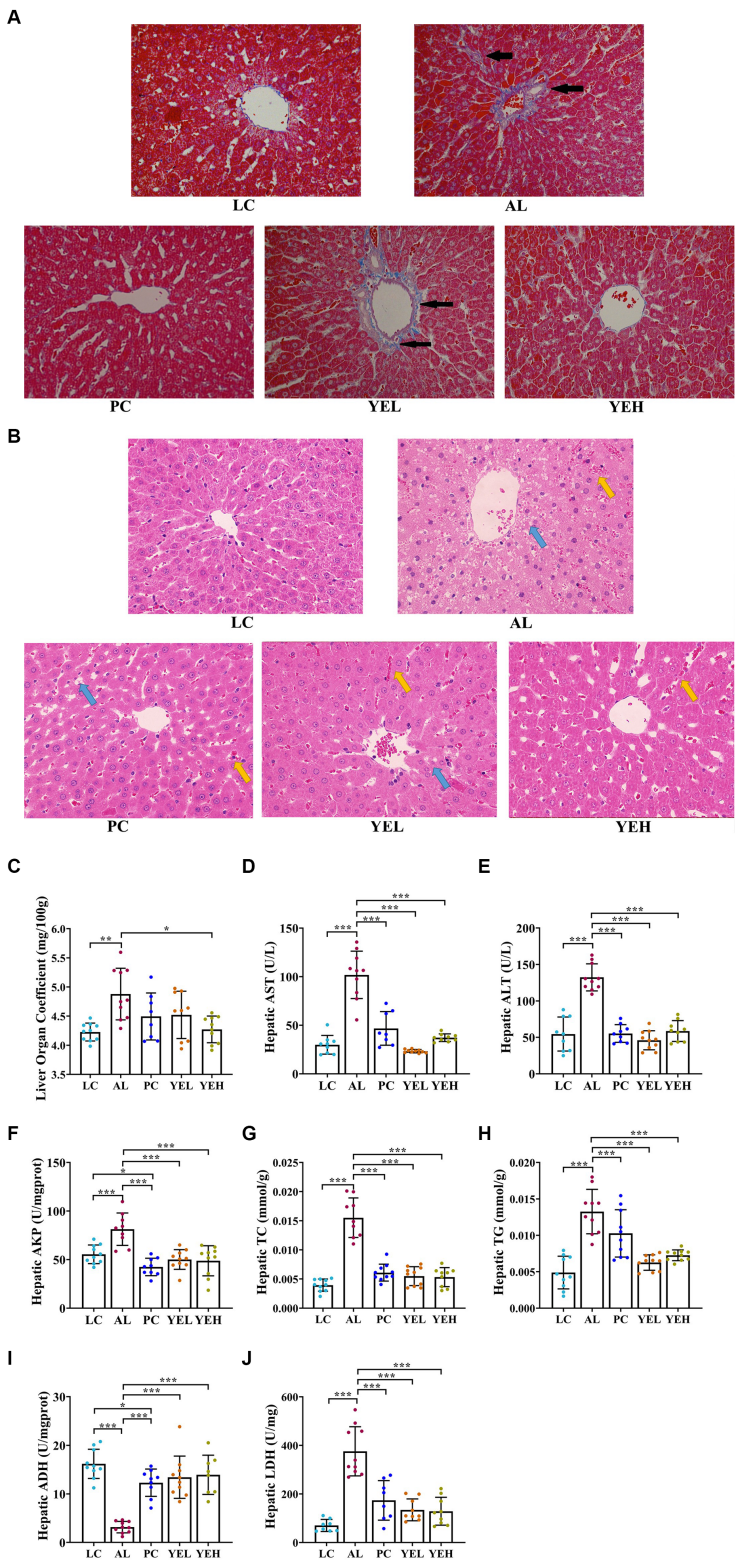
Effect of yeast extract on alcohol-induced liver injury. **(A)** Histopathological changes of liver sections stained with Masson (400× magnification), black arrow: liver tissue fibrosis. **(B)** Histopathological changes of liver sections stained with H&E (400× magnification), yellow arrow: inflammatory exudation; blue arrow, steatosis. **(C)** The levels of liver organ coefficient. **(D)** The hepatic AST levels. **(E)** The hepatic ALT levels. **(F)** The hepatic AKP levels. **(G)** The hepatic TC levels. **(H)** The hepatic TG levels. **(I)** The hepatic ADH contents. **(J)** The hepatic LDH contents. All data were expressed as mean ± SD (*n* = 8–10), ^*^p < 0.05, ^**^p < 0.01, ^***^p < 0.001.

To explore the effects of YE on ALD, H&E and Masson staining were performed to check the histomorphological changes that were induced by alcohol. The hepatocytes arranged neatly without obvious pathological changes in the LC group, but there was irregular arrangement of hepatocytes, diffuse steatosis, and a large number of inflammatory cell infiltration in AL group (Figure 1B). According to Masson staining, there were more parts that were dyed blue in the AL group, suggesting a more serious case of fibrosis (Figure 1A). Compared with the AL group, the pathological changes observed in the liver were alleviated in the PC, YEL, and YEH groups, with YEH exhibiting the most significant improvement (Figures 1A,B). In comparison to the AL group, a remarkable decrease in the liver organ coefficient was observed in the YEH group (*p* < 0.05, Figure 1C), whereas no significant reduction was noted in the PC and YEL groups. Biochemical analysis demonstrated that the levels of hepatic ALT, AST, and AKP, which are diagnostic markers of liver injury, remarkably increased in the AL group compared to the LC group. However, the administration of YE and Silymarin effectively inhibited the increase (Figures 1D–F). The administration of YE also reduced the accumulation of TC and TG (*p* < 0.01, Figures 1G,H). Additionally, high doses of YE significantly decreased LDH levels (*p* < 0.01, Figure 1J). Furthermore, YE administration effectively prevented the decrease in ADH levels (*p* < 0.001, Figure 1I).

### 3.2. YE inhibited alcohol-induced oxidative stress

**Figure 2:**
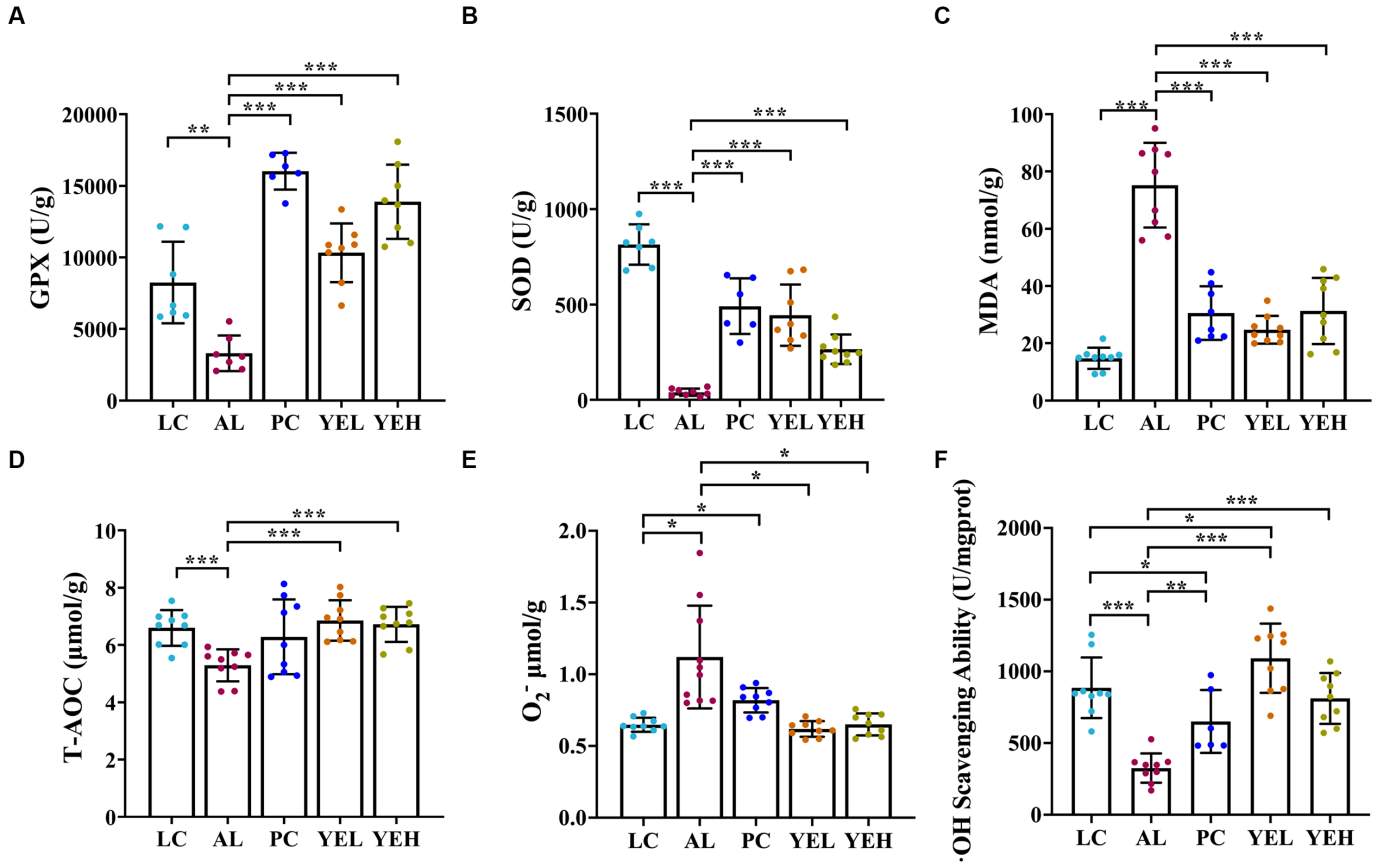
Effect of yeast extract on oxidative stress in alcohol-induced liver injury in rats. **(A)** The levels of GPX. **(B)** The levels of SOD. **(C)** The levels of MDA. **(D)** The levels of T-AOC. **(E)** The levels of O_2_^−^. **(F)**·OH scavenging ability. Data were expressed as mean ± SD (*n* = 6–9), ^*^p < 0.05, ^**^p < 0.01, ^***^p < 0.001.

The levels of T-AOC, GPX, SOD and·OH scavenging ability remarkably decreased in the AL group than in the LC group (Figures 2A,B,D,F). On the other hand, the levels of MDA and O_2_^−^ exhibited opposing trends (*p* < 0.05, Figures 2C,E). It is noteworthy that the groups with the YE intervention exhibited strong antioxidant activity by resisting alcohol-induced liver damage, which showed good protective effects (*p* < 0.05, Figures 2A–F). However, there were no significant changes in the levels of T-AOC and O_2_^−^ after treatment with Silymarin (*p* > 0.05, Figures 2D,E). The highest level of ·OH scavenging ability was observed in the YEL group (*p* < 0.05, Figure 2F).

### 3.3. YE reversed alcohol-induced inflammatory response

**Figure 3:**
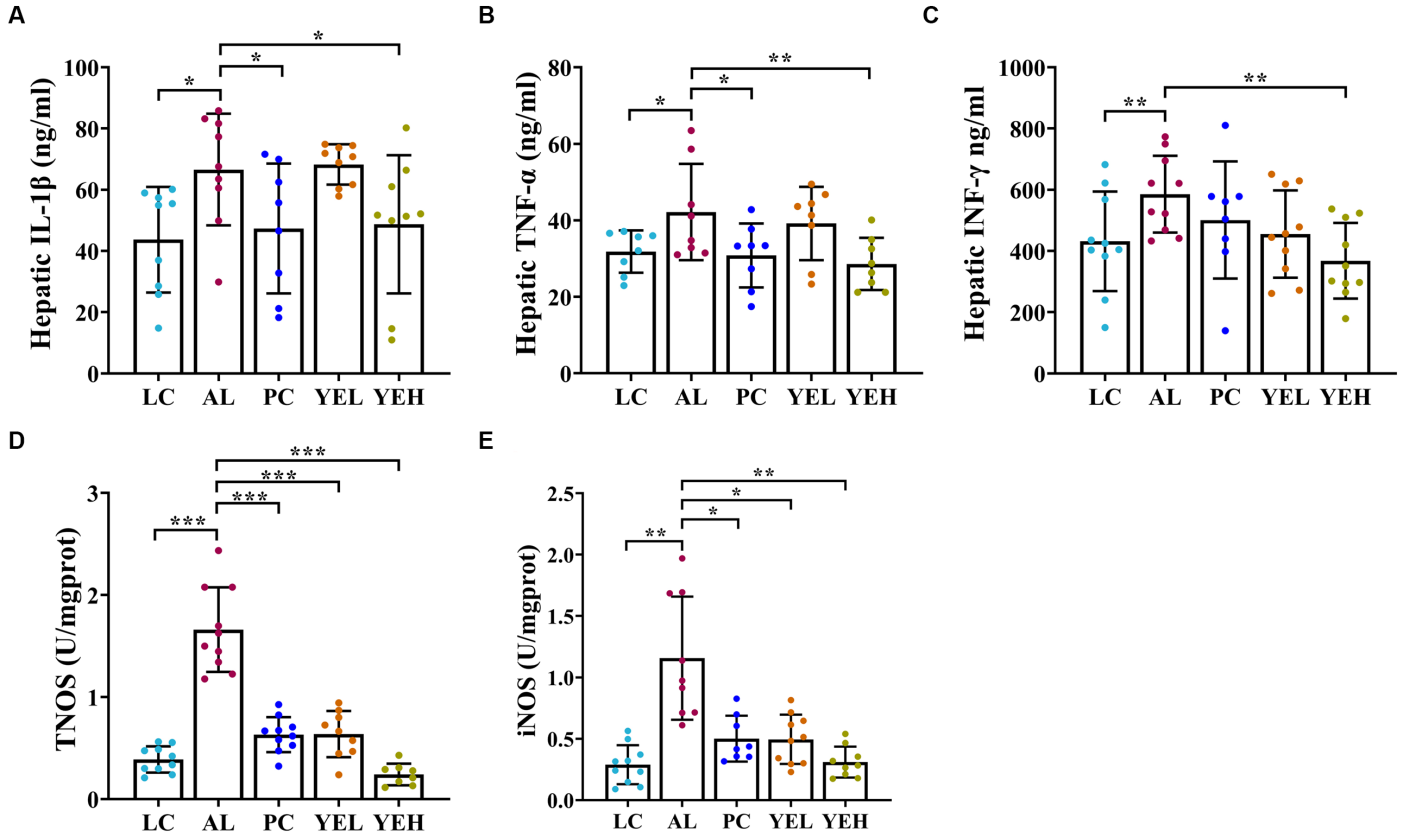
Effect of yeast extract on inflammatory response in alcohol-induced liver injury in rats. **(A)** The levels of IL-1β. **(B)** The levels of TNF-α. **(C)** The levels of IFN-γ. **(D)** The levels of TNOS. **(E)** The levels of iNOS. Data were expressed as mean ± SD (*n* = 8–10), ^*^p < 0.05, ^**^p < 0.01, ^***^p < 0.001.

Some studies have reported that excessive or chronic alcohol use can trigger an inflammatory response in the liver (Xiao et al., 2020; Hyun et al., 2021). As shown in Figure 3, alcohol ingestion significantly increased the levels of IL-1β, TNF-α, IFN-γ, iNOS, and TNOS in the liver of rats (*p* < 0.05, Figures 3A–E). Compared with the AL group, administration with high-dosage YE inhibited alcohol-induced inflammatory response, and this was consistent with LC group (*p* < 0.05, Figures 3A–E). However, the levels of IL-1β, TNF-α, and IFN-γ in the YEL group and those of IFN-γ in the Silymarin group did not exhibit obvious changes (*p* > 0.05, Figures 3A–C). These findings suggested that YE intervention can reduce the levels of inflammatory factors and alleviate alcohol-induced liver damage. High dosage of YE appear to provide superior protective effects.

### 3.4. Effects of YE intervention on gut microbiota

ALD is closely associated with the gut microbiota. Long-term consumption of alcohol can substantially alter the structure of the gut microbiota in ALD patients (Bishehsari et al., 2017; Wang et al., 2021).To characterize alterations in gut microbiota, fecal samples were subjected to 16S rRNA gene sequencing. The analysis revealed specific and shared OTUs among the five groups of mice (Supplementary Figure S1A). In comparison to the LC group, all experimental groups exhibited an increase in both the Shannon and Simpson diversity index (Supplementary Figures S1B,C). The Weighted Unifrac analysis demonstrated that the β-diversity of gut microbiota in the PC, YEL, and YEH groups was significantly distinct from that of the AL group, but closer to that of the LC group (Supplementary Figures S1D,E). These results suggest that YE intervention could reverse alcohol-induced dysbiosis of gut microbiota in rats.

**Figure 4:**
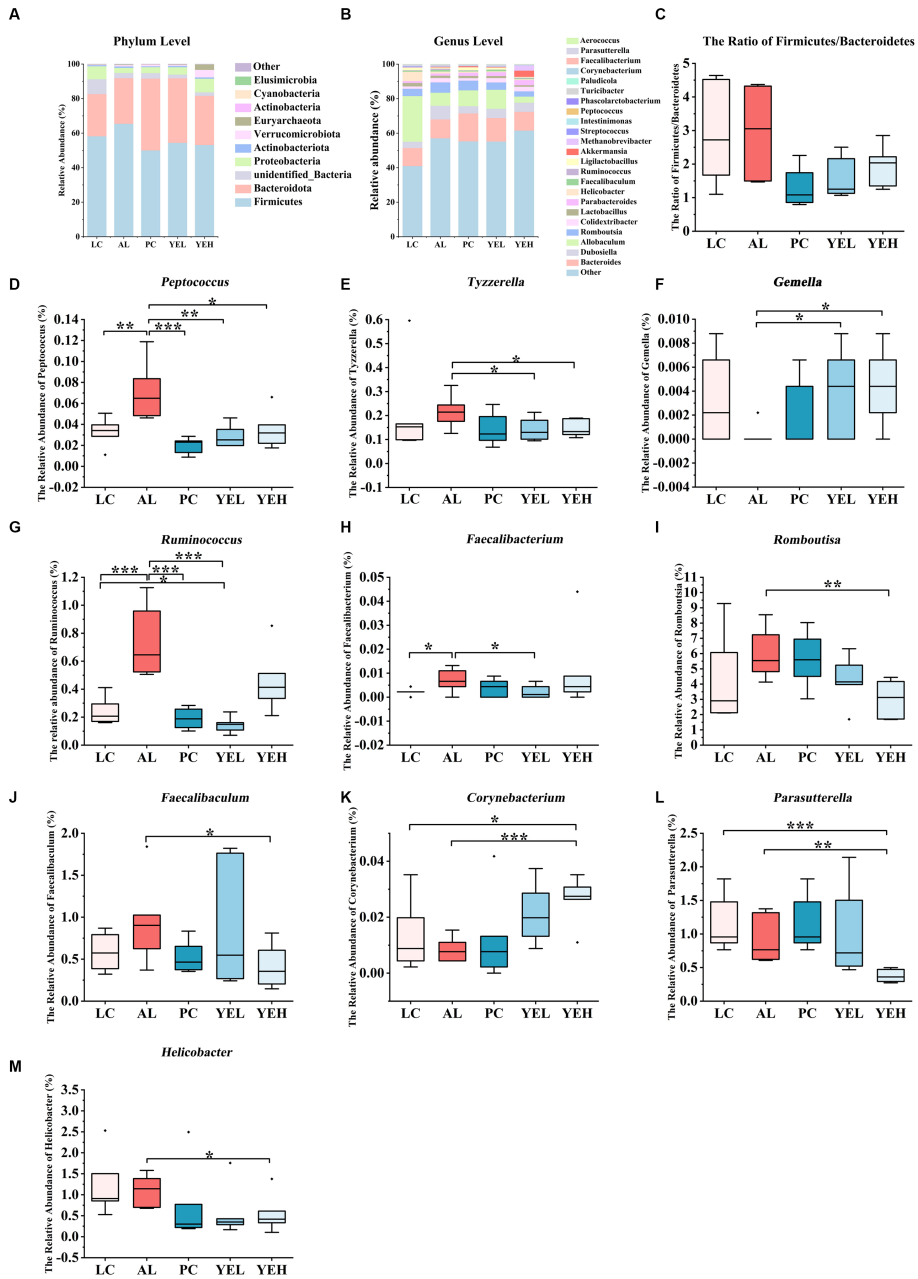
Yeast extract intervention altered the gut microbiota structure of alcohol-induced liver injury in rats. **(A)** Bacterial taxonomic composition at phylum level. **(B)** Bacterial taxonomic composition at genus level. **(C)** The ratio of Firmicutes to Bacteroidetes (F/B). **(D)** The relative abundance of *Peptococcus*. **(E)** The relative abundance of *Tyzzerella.* **(F)** The relative abundance of *Gemella*. **(G)** The relative abundance of *Ruminococcus*. **(H)** The relative abundance of *Faecalibacterium*. **(I)** The relative abundance of *Romboutsia.* **(J)** The relative abundance of *Faecalibaculum*. **(K)** The relative abundance of *Corynebacterium*. **(L)** The relative abundance of *Parasutterella*. **(M)** The relative abundance of *Helicobacter*, *n* = 6, ^*^p < 0.05, ^**^p < 0.01, ^***^p < 0.001.

The structure of the gut microbiome was investigated at both phylum and genus levels (Figures 4A,B). Bacteroidetes and Firmicute were the most abundant at the phylum level in all groups. However, when compared with the AL group, the ratio of Firmicutes to Bacteroidetes (F/B) was reduced in the other groups, though the change was not significant (Figure 4C). On the other hand, the population of Proteobacteria was significantly elevated in both the YEH and PC groups (*p* < 0.05), compared to the AL group. At the genus level, intervention with YEL and YEH resulted in a decrease in the relative abundance of *Peptococcus* and *Tyzzerella,* while an increase in the relative abundance of *Gemella* (Figures 4D–F), compared to the AL group was observed. In the YEL group, the relative abundance of *Ruminococcus* and *Faecalibacterium* was significantly decreased (Figures 4G,H). It was also noted that the administration of YEH resulted in a significantly reduced relative abundance of *Faecalibaculum*, *Helicobacter, Parasutterella* and *Romboutsia* compared to the AL group, while the *Corynebacterium* remarkably increased (Figures 4I–M).

**Figure 5:**
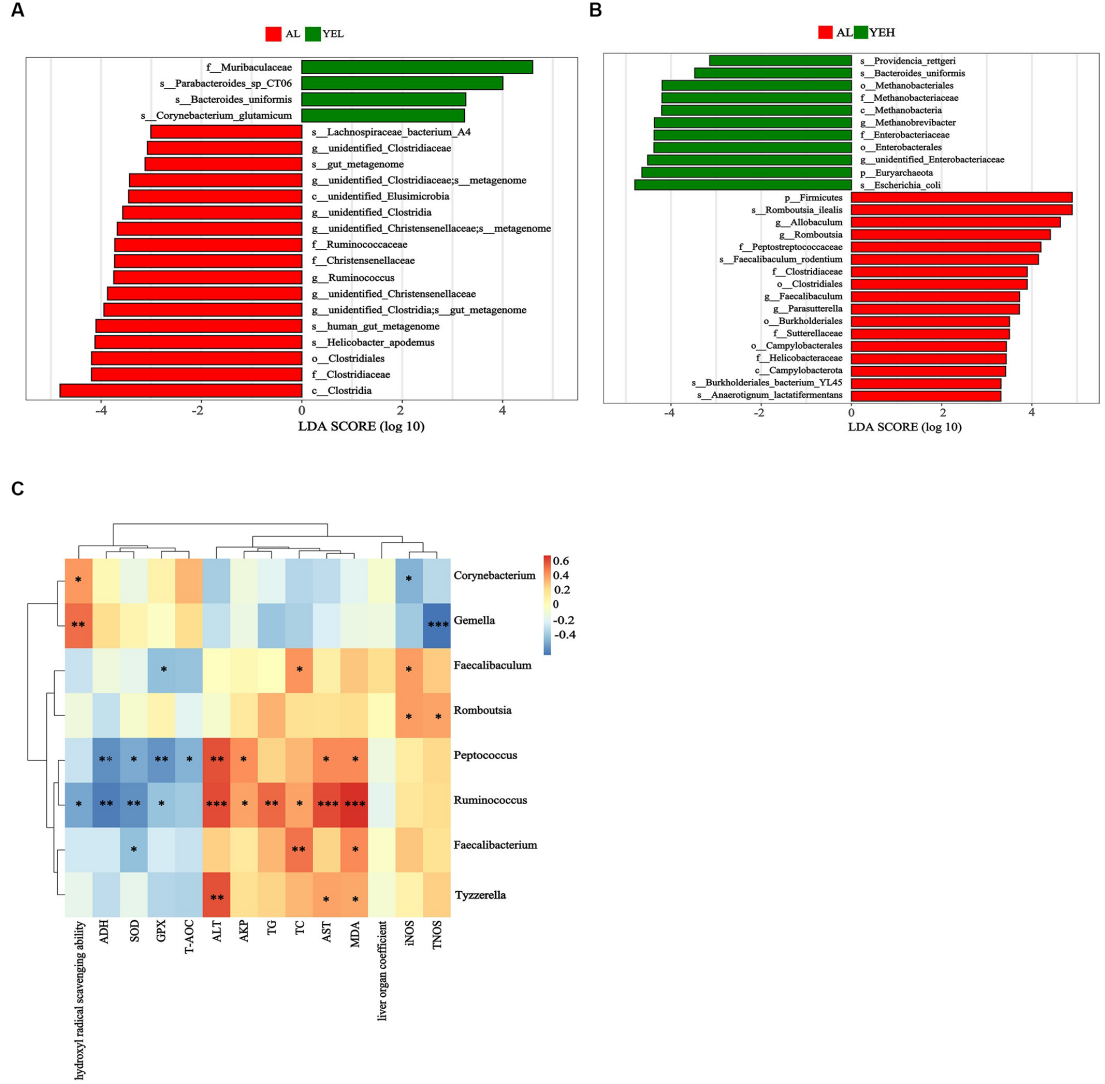
Linear discriminant analysis (LDA) and the correlation between genera and liver related indicators. **(A)** Linear discriminant analysis between AL group and YEL group. **(B)** Linear discriminant analysis between AL group and YEH group. **(C)** Spearman’s correlation between the liver related indicators and the relative abundance of significantly different genera, *n* = 6, ^*^p < 0.05, ^**^p < 0.01, ^***^p < 0.001. Red squares represent positive correlation and blue squares represent negative correlation. The darker the red, the greater the correlation coefficient, and the darker the blue, the smaller the correlation coefficient.

Linear discriminant analysis (LDA) and LEfSe analysis were used to identify biomarkers with significant differences. Based on LDA scores, the relative abundance of *Ruminococcus* was significantly higher in the AL group compared to the YEL group (Figure 5A). In addition, *Romboutsia, Faecalibaculum*, and *Parasutterella* were found to be more abundant in the AL group than in the YEH group (Figure 5B). The alterations in gut microbiota have a certain degree of correlation with some physicochemical indicators that are related to liver injury. As shown in Figure 5C, the genera such as *Tyzzerella* and *Peptococcus* decreased after YE intervention, and these positively correlated with ALT, AST, and MDA. In addition, positive correlation was observed between *Peptococcus* and AKP. However, negative correlation was noted between *Peptococcus* and ADH, SOD, GSH, and T-AOC. After YE intervention, *Gemella* increased, while *Corynebacterium* levels only increased in the YEH group. Both genera showed significant positive correlations with hydroxyl radical scavenging ability, but significant negative correlations with TNOS were observed (Figure 5C). In the YEL group, *Faecalibacterium* and *Ruminococcus* significantly decreased. *Faecalibacterium* showed a positive correlation with MDA and TC, but a negatively correlation with SOD. On the other hand, the abundance of *Ruminococcus* positively correlated with ALT, AST, AKP, TC, TG, and MDA, while it was negatively associated with levels of ADH, SOD, GPX, and hydroxyl radical scavenging ability (Figure 5C). The genera *Romboutsia* and *Faecalibaculum* decreased in the YEH intervention group. *Romboutsia* exhibited a significant positive correlation with TNOS and iNOS, while *Faecalibaculum* showed a positive correlation with TC and iNOS, but a negative association with GPX (Figure 5C).

### 3.5. Yeast extract altered metabolic profiles

**Figure 6:**
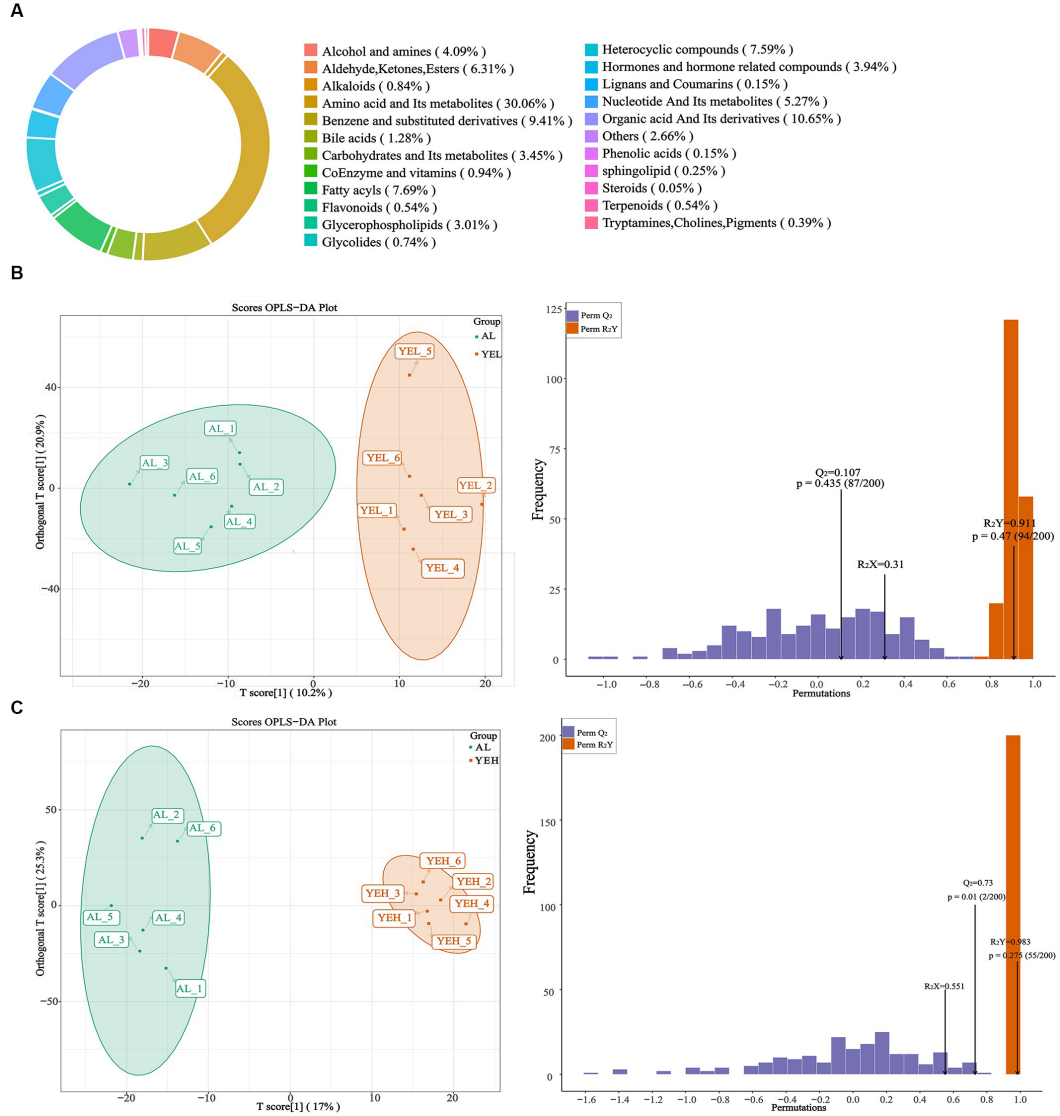
Effects of yeast extract on metabolic profiling. **(A)** Composition of all metabolites. **(B)** The orthogonal partial least-squares discriminant analysis (OPLS-DA) of AL and YEH groups. **(C)** OPLS-DA of AL and YEH groups.

**Figure 7:**
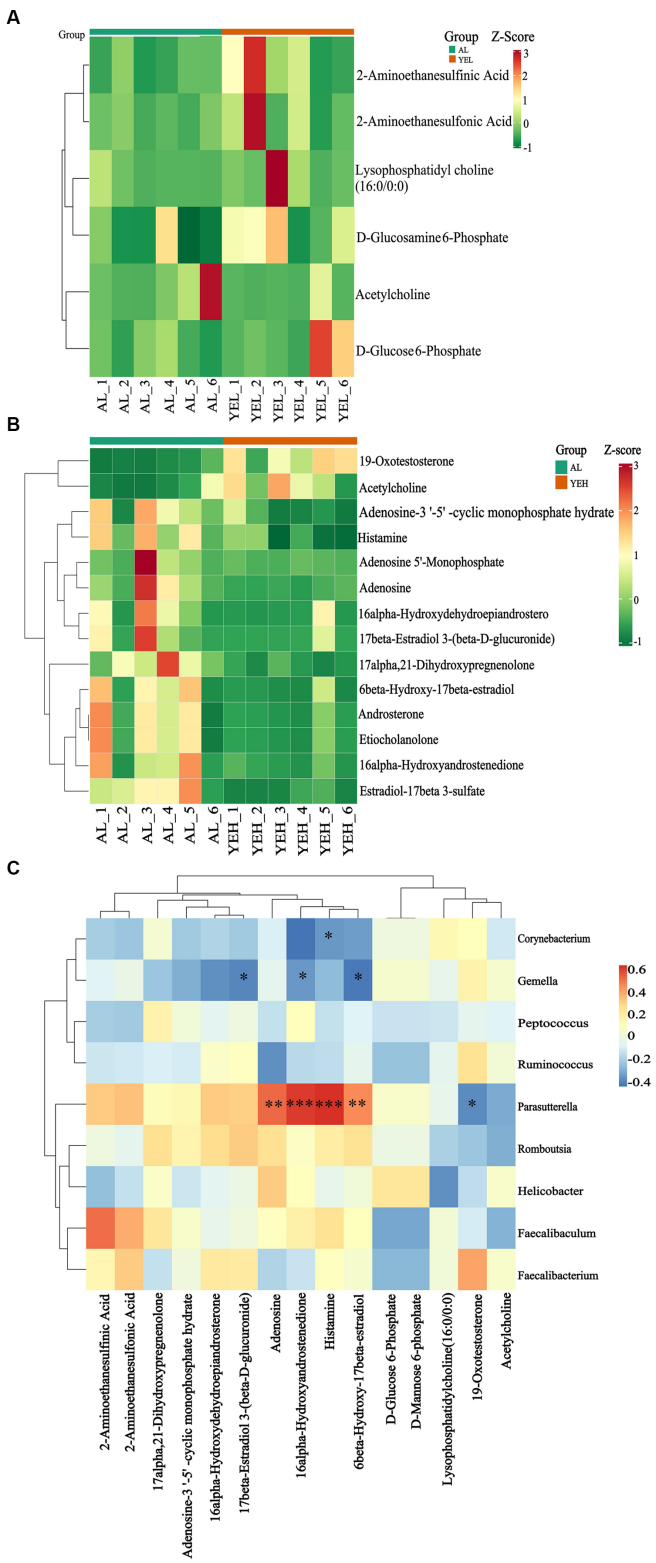
Effects of yeast extract on metabolic profiles. **(A)** Heatmap of differential metabolites in AL and YEL groups. **(B)** Heatmap of differential metabolites in AL and YEH groups. **(C)** Spearman’s correlation between the relative abundance of significantly different genera and differential metabolites. Red squares represent positive correlation while blue squares represent negative correlation. The darker the red, the greater the correlation coefficient, and the darker the blue, the smaller the correlation coefficient, *n* = 6.

To further explore and understand the effects of altered gut microbiota on metabolite changes, a total of 2029 metabolites were detected through the extensive targeted metabolomics analysis (Figure 6A). According to the orthogonal partial least-squares discriminant analysis (OPLS-DA), metabolites in the YEH group could be well separated from those in the AL group (Q^2^ > 0.5) (Figure 6C). Based on the threshold of fold change ≥2 or fold change ≤0.5 and VIP > 1, 201 differential metabolites were identified between the AL and YEL groups, while 470 differential metabolites were found between AL and YEH groups. By combining pathways with significant enrichment in KEGG and HMDB databases, it was discovered that among the samples of AL and YEH, 14 metabolites were significantly different (two up-regulated metabolites and 12 down-regulated metabolites). Additionally, there were six remarkedly different metabolites (five up-regulated metabolites and one down-regulated metabolite) among the samples of AL and YEL groups. The heatmaps of specific metabolic difference substances are shown (Figures 7A,B).

To explore the relationship between the co-regulation of gut microbes and metabolites, the Spearman correlation analysis was performed (Figure 7C). *Gemella* levels were found to exhibit notable negative correlation with 17β-estradiol 3-(β-D-glucuronide) concentrations. *Parasutterella* positively correlated with histamine and adenosine, while *Corynebacterium* inversely correlated with histamine.

**Figure 8:**
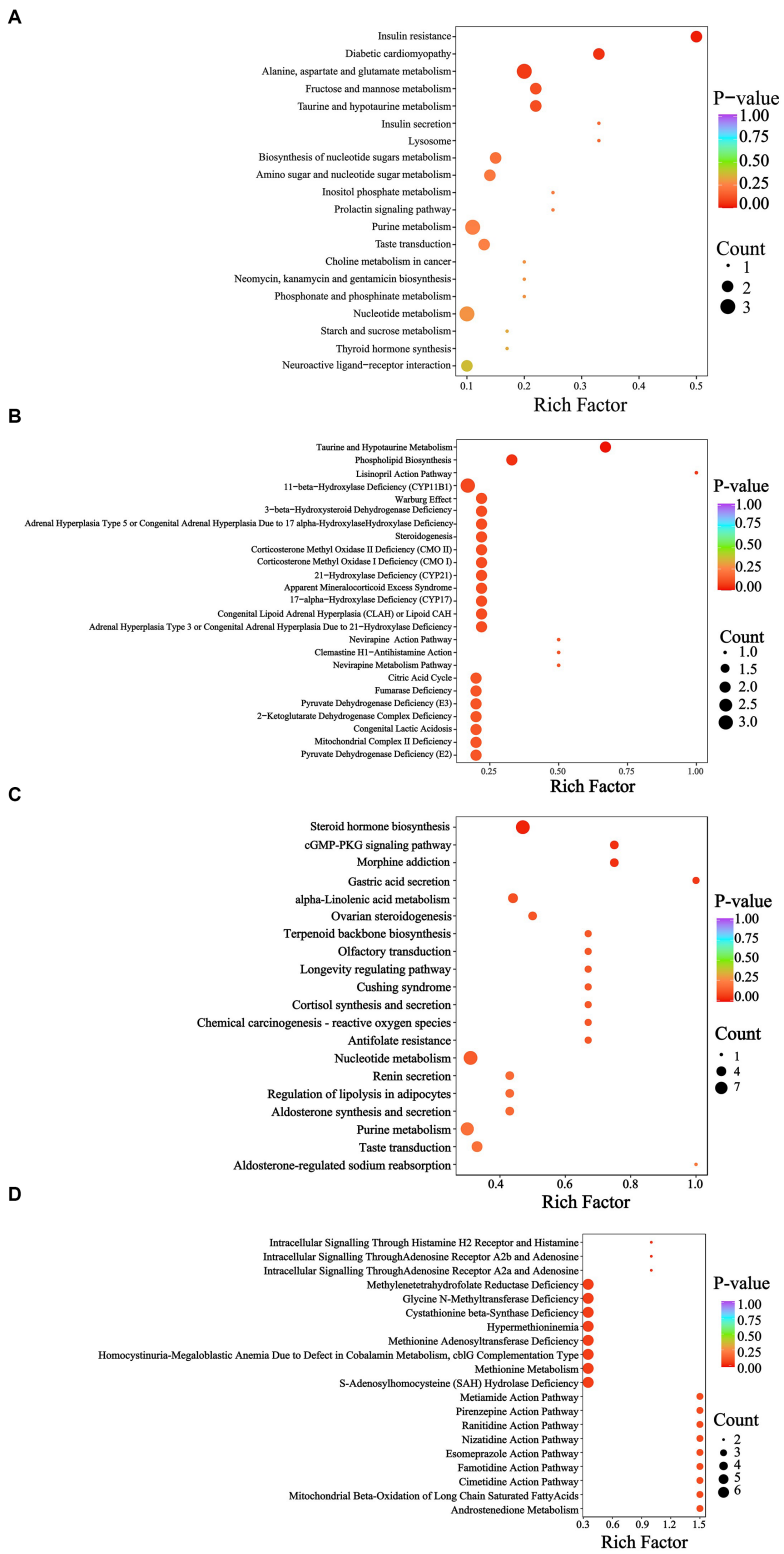
Metabolic pathway analysis of differential metabolites. **(A)** KEGG enrichment pathways based on the differential metabolites (AL and YEL). **(B)** HMDB enrichment pathways based on the differential metabolites (AL and YEL). **(C)** KEGG enrichment pathways based on the differential metabolites (AL and YEH). **(D)** HMDB enrichment pathways based on the differential metabolites (AL and YEH).

According to the KEGG database, the YEL notably affected insulin resistance (Figure 8A), while the HMDB database indicated that the intervention of the YEL significantly affects two major metabolic pathways: taurine and hypotaurine metabolism, as well as phospholipid biosynthesis. According to the KEGG database, there are significant differences in steroid hormone biosynthesis, morphine addiction, cGMP-PKG signaling pathway, and gastric acid secretion between the AL and YEH groups (Figure 8C). Based on the HMDB database, YEH significantly impacted three metabolic pathways: intracellular signaling via adenosine receptor A2a and adenosine, intracellular signaling through adenosine receptor A2b and adenosine, and intracellular signaling mediated histamine receptor H2 and histamine (Figure 8D).

## 4. Discussion

YE has strong antioxidant and anti-inflammatory properties. Oxidative stress and inflammation are important mechanisms that greatly contribute to alcoholic liver injury. Yeast extract primarily consists of yeast cell wall, yeast protein, and yeast nucleotide. The presence of β-glucan in the yeast cell wall has been scientifically demonstrated to enhance the activity of immune cells, thereby boosting immunity (Zhang et al., 2018). Additionally, yeast nucleotide has been found to mitigate the adverse effects of *Eimeria* on the intestinal tract (Leung et al., 2019). In this study, we hypothesized that the YE could exert protective effects against liver injury. We established an animal model for alcoholic liver injury. Related physicochemical indicators, as well as the response of the gut microbiota and metabolites were analyzed to verify our hypothesis.

As expected, the levels of AST, ALT, AKP (Figures 1D–F), TC and TG (Figures 1G,H) in the rats of the AL group significantly increased. Hepatocyte damage can be assessed by the rise in ALT and AST levels (Giannini et al., 2005; Ozer et al., 2008), while the increase of AKP might suggest that cholestasis could occur in the liver (Ramaiah, 2007). Chronic alcohol consumption can lead to hepatic lipid metabolism disorders, resulting in a rise of TG and total TC levels (Xu et al., 2018). After the YE intervention, the levels of the above-mentioned indicators were significantly reduced in both the YEL and the YEH groups. This indicated that YE can effectively alleviate alcohol-induced liver cell dysfunction and promote lipid metabolism.

Oxidative stress is an imbalance between antioxidants and oxidation in the body, with the former being significantly lower. Ethanol consumption depletes a substantial amount of antioxidant enzymes (such as SOD or GPX) and generates an abundance of ROS, which ultimately causes damage. The intervention of the YE can markedly increase GPX, SOD and T-AOC (Figures 2A,B,D), while significantly decreasing MDA (Figure 2C), thereby effectively reducing oxidative stress (Yuan et al., 2018; Gao et al., 2022).

After the YE and silymarin interventions, the levels of TNOS and iNOS were significantly reduced (Figures 3D,E). TNOS and iNOS are crucial indicators of inflammatory injury so reducing the expression levels of their related genes can lower the inflammatory response (Lee et al., 2016). Continuous intake of ethanol induces the production of iNOS (Davis and Syapin, 2004), an isoform of nitric oxide synthase which oxidizes L-arginine to L-citrulline and generates nitric oxide (Chen et al., 2015). The nitric oxide combines with the superoxide anion to form an oxidant that causes lipid peroxidation, which damages the body (Zhu et al., 2023). In addition, the intervention of YEH significantly reduced the levels of IL-1β, TNF-α, and IFN-γ (Figures 3A–C), with effects that were even better than those that were observed in the silymarin intervention group. LPS that are produced by intestinal bacteria can stimulate Kupffer cells to secrete TNF-α. In addition, rats with lower IL-1β levels exhibited a significant reduction in the transition from steatosis to steatohepatitis and liver fibrosis, suggesting that IL-1β also plays a key role in liver injury (Tilg and Moschen, 2011). The results demonstrate that the intervention of the YE can effectively improve the alcohol-induced liver oxidative stress and inflammatory response, with the YEH exhibiting better anti-inflammation efficacy.

The 16S rRNA sequencing results showed that the *β*-diversity of LC, YEL, and YEH was significantly higher than that of the AL group (Supplementary Figures S1D,E), with no significant difference among the three groups. Studies indicated that ethanol can significantly decrease the level of β-diversity, which is closely related to alterations in the gut microbiota (Piacentino et al., 2021; Xia et al., 2021). In terms of gut microbiota composition, the ratio of Firmicutes to Bacteroidetes in the YE group was lower than that in the alcohol-induced model group (Figure 4C). This ratio positively correlated with the extent of gut microbiota disturbance (Shao et al., 2018; Belancic, 2020; Jasirwan et al., 2021). This indicated that the YE has certain positive regulatory effects on the gut microbiota.

At the genus level, the intervention of the YEL could significantly reduce the relative abundance of *Ruminococcus* and *Faecalibacterium* (Figures 4G,H). Studies have shown that the relative abundance of *Ruminococcus* in the intestinal tract of obese and non-alcoholic fatty liver children is significantly higher than that of healthier children (Del Chierico et al., 2017). In addition, *Ruminococcus* has also been confirmed to be positively associated with liver function indicators like alanine aminotransferase (AST) and aspartate aminotransferase (ALT) (Jiao et al., 2022; Quan et al., 2022). The relative abundance of *Faecalibacterium* in patients with alcoholic hepatitis significantly increased (Philips et al., 2019).

In the YEH intervention group, the relative abundances of *Faecalibaculum*, *Helicobacter, Romboutsia,* and *Parasutterella* were significantly lower than those in the alcohol-induced model group (Figures 4I,J,L,M). According to the analysis of the correlation between intestinal microbes and liver injury factors, *Romboutsia* and *Faecalibaculum* positively correlated with TNOS and iNOS. Previous studies have shown that alcohol intake can significantly increase the relative abundance of *Romboutsia*, accompanied by an increase in the mRNA of fibrosis-related markers (Yu et al., 2020). It was also reported that the relative abundance of *Helicobacter* increases in mouce with alcohol feeding (Philips et al., 2022) and positively correlate with the extent of the oxidative stress that is caused by chronic inflammation (Hardbower et al., 2013). By comparing the differences in gut microbiota between nonalcoholic fatty liver rats induced by high-fat diet and normal diet rats (Zhuge et al., 2022), it was found that *Helicobacter*, *Romboutsia* and *Faecalibaculum* were enriched in the high-fat diet group, further characterized by the presence of hepatic oxidative stress, fibrosis, and intestinal inflammation. Additionally, *Parasutterella* was positively associated with intestinal inflammation and irritable bowel disorder (Huang et al., 2017; Chen et al., 2018). In this study, the intervention of YEH significantly reduced the relative abundance of the above-mentioned microorganisms, indicating that it can alleviate the liver inflammatory response and lipid metabolism disorder that is caused by alcohol. This happens through the downregulation of the levels of *Helicobactere*, *Romboutsia, Faecalibaculum,* and *Parasutterella*, thereby exerting a protective effect on the liver.

The intervention of YEH can also significantly increase the relative abundance of *Corynebacterium* (Figure 4K). Both YEL and YEH interventions can reduce the relative abundance of *Peptococcus* and *Tyzzerella* (Figures 4D,E). Studies have reported that the relative abundance of *Corynebacterium* and *Tyzzerella* is significantly decreased in people with non-alcoholic fatty liver disease (Kordy et al., 2021; Zhang et al., 2021). Besides, a mouse experiment targeting liver fat accumulation showed a significant increase in the relative content of *Tyzzerella*. The contents of TC, TNF- α, IFN- γ, and IL-1β are positively correlated with *Tyzzerella*. This further indicates that YE can enhance antioxidant ability in the liver, which reduces alcohol-induced liver damage.

According to KEGG and HMDB databases, there were enriched pathways between the AL and YEL groups and between the AL and YEH groups. Compared with the AL group, YEL intervention significantly up-regulated the levels of D-mannose-6-phosphate and D-glucose-6-phosphate (Figure 7A). D-glucose-6-phosphate is a product from the phosphorylation of glucose by glucokinase. If it accumulates in the liver for a long time, it can promote triglyceride synthesis and lead to lipid accumulation in the liver (Rajas et al., 2019). In addition, low doses of YE can also affect the metabolism of taurine and hypo taurine (Figure 8B), resulting in a significant increase in the content of taurine (2-aminoethane sulfonic acid). This substance is widely used as a dietary supplement, which can reduce the expression of TNF-α, IL-6, and IL-1β mRNA and reduce the inflammatory response (Shi et al., 2021). Taurine can also increase the activity of SOD and MDA, thereby reducing oxidative stress that is caused by alcohol (Murakami et al., 2018).

Lysophosphatidylcholine (16:0/0:0) (Figure 7A), which is present in the phospholipid biosynthesis pathway, was also significantly increased. It has been confirmed to alleviate lung injury of acute respiratory distress syndrome by down-regulating the mRNA expression of inflammatory factors such as TNF-α, IL-6, and IL-1β, as well as by reducing neutrophil infiltration (Du et al., 2022). This suggests that lysophosphatidylcholine (16:0/0:0) has certain anti-inflammatory effects. However, in the YEL group, the content of acetylcholine (Figures 7A,B) in the pathway was significantly lower than that in the AL group, while the levels of this substance in the YEH group were significantly higher (Figure 7B). Acetylcholine can alleviate colonic inflammation by promoting the secretion of Interleukin 10 (IL-10) and down-regulating the mRNA expression of TNF-α, IFN-γ, and IL-1β. Thus, the YEH group has better anti-inflammatory properties.

Observations from this study showed that the YEH intervention tends to downregulate the secretion of gastric acid secretion (Figure 8C). Some studies have reported that long-term alcohol consumption can damage the gastric mucosal barrier defense system and reduce gastric mucosa’s ability to resist the invasion of gastric acid, bile, and various digestive enzymes. Excessive gastric acid can cause mucosal damage (Ning et al., 2012; Wu et al., 2021). At the same time, gastric acid inhibitors (Zhang et al., 2016) have been confirmed to reduce the damage of alcohol to the stomach. Therefore, high doses of yeast extract have a certain protective effect on the stomach. In addition, YEH was able to reduce the content of histamine in this pathway, which mediates GI tract damage that is induced by alcohol (Zimatkin and Anichtchik, 1999). Studies in mouse models have found that the oral administration of histamine can cause gut microbiota disorder, oxidative stress, inflammatory response (Luo et al., 2022), and cause liver fibrosis (Kennedy et al., 2020). In morphine addiction and cGMP-PKG signaling pathways, high doses of yeast extract modulate both pathways by reducing the levels of adenosine and 5′ -adenine nucleotide (Figure 7B). The acetaldehyde that is produced by alcohol metabolism can inhibit adenosine reabsorption (Nagy et al., 1990) and is associated with the level of adenosine and cGMP-PKG signaling. ATP- binding produces adenosine (Pardo et al., 2013; Purnell et al., 2023), which, in excess, inhibits respiration (Dunwiddie and Masino, 2001) and promotes liver fibrosis (Fausther, 2018). Also, when 5 ‘-monophosphate was injected, the expression of genes related to fatty acid metabolism in the liver was significantly inhibited (Kondo et al., 2020). However, histamine and adenosine levels positively correlated with *Parasutterella*, while *Corynebacterium* inversely correlated with histamine (Figure 7C). While *Corynebacterium* numbers showed a significant positive correlation with ·OH clearance, those of *Parasutterella* were shown to be positively correlated with intestinal inflammation and irritability (Huang et al., 2017; Chen et al., 2018). Therefore, the antioxidant activity of YE is closely related to the gut microbiota and their metabolites.

## 5. Conclusion

In this study, the administration of different concentrations of yeast extract to ALD rats could alleviate the alcohol-induced liver hepatoxicity, oxidative stress, and inflammatory response. Compared with the YEL, the YEH inhibited the inflammatory response to a greater extent exhibiting even better results than the positive drug group. Yeast extract can also improve gut microbiota disorders and regulate their metabolic products in ALD rats, thus alleviating liver oxidative stress and inflammatory damage through the “liver-intestine” axis. It is important to note that high-dose yeast extract has a greater effect on metabolic products. However, there are some limitations associate with study that can be addressed in future research. First, although we have shown that yeast extract has a protective effect on alcohol-induced liver damage, it is a complex mixture whose functional components need to be further explored. Second, we observed that yeast extract intervention had impacts on some gut microbiota and their metabolites. However, further exploration of the functional validation of both is still required to better explain the mechanism through which yeast extract affects alcohol-induced liver damage.

## Data availability statement

The datasets presented in this study can be found in online repositories. The names of the repository/repositories and accession number(s) can be found below: NCBI – PRJNA975355.

## Ethics statement

The animal study was reviewed and approved by Medical Ethics Committee of School of Public Health, Lanzhou University.

## Author contributions

DL, ZL, and YL designed this study. ZL, MW, YW, LF, and SW performed experimental work. DL and YL revised methodology and provided resources. ZL analyzed data and wrote the first draft of the manuscript. DL, YL, HL, XL, YZ and DG supervised and interpreted the experimental data, and critically revised the manuscript. All authors contributed to the article and approved the submitted version.

## Funding

This study was supported by the Science and Technology Department of Gansu Province, China (20YF3WA014).

## Publisher’s note

All claims expressed in this article are solely those of the authors and do not necessarily represent those of their affiliated organizations, or those of the publisher, the editors and the reviewers. Any product that may be evaluated in this article, or claim that may be made by its manufacturer, is not guaranteed or endorsed by the publisher.

## Conflict of interest

The authors declare that the research was conducted in the absence of any commercial or financial relationships that could be construed as a potential conflict of interest.

## Footnotes

^1^http://www.arb-silva.de/

^2^https://www.kegg.jp

^3^http://www.hmdb.ca/

## Abbreviations

ADH: Alcohol Dehydrogenase
AKP: Alkaline phosphatase
ALD: Alcohol liver disease
ALT: Alanine aminotransferase
AST: Aspartate aminotransferase
GPX: Glutathione peroxidase
IL-1β: interleukin-1β
INF-γ: interferon-γ
iNOS: inducible nitric oxide synthase
LDH: Lactate dehydrogenase
MDA: Malondialdehyde
OH Scavenging Ability: Hydroxyl Radical Scavenging Ability
SOD: Superoxide dismutase
T-AOC: Total antioxidant capacity
TC: Total Cholesterol
TG: Triglyceride
TNF-α: tumor necrosis factor-α
TNOS: total nitric oxide synthase.
