# Supplementary figures and images for: Protective effects of yeast extract against alcohol-induced liver injury in rats
Source: Front Microbiol. 2023 Jul 20;14:1217449. doi: 10.3389/fmicb.2023.1217449 (PMC10399763; doi:10.3389/fmicb.2023.1217449)

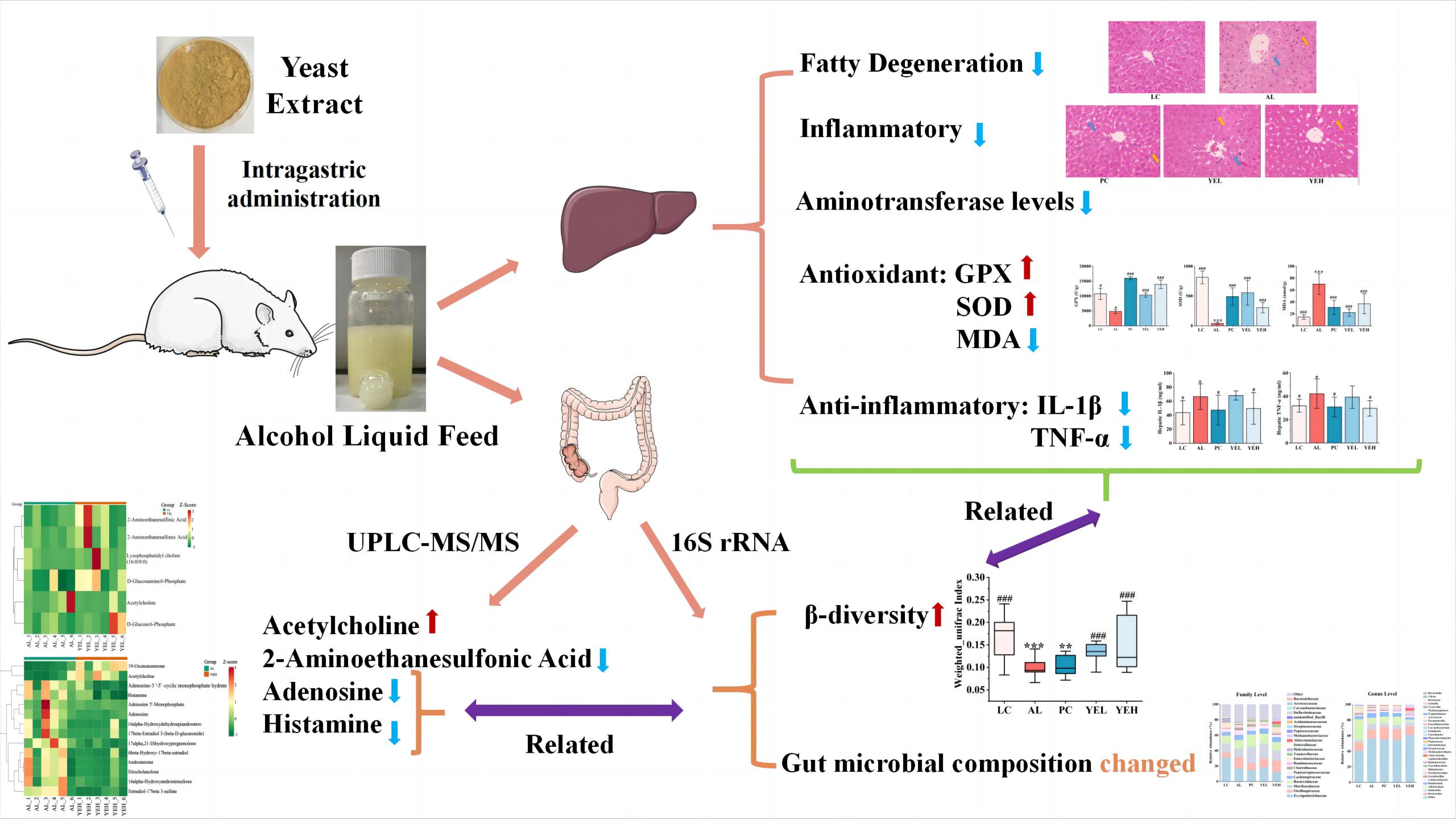

Supplement: Supplementary file 1 [file Image_1.JPEG]
