# Supplementary material for: Protective effects of yeast extract against alcohol-induced liver injury in rats
Source: Front Microbiol. 2023 Jul 20;14:1217449. doi: 10.3389/fmicb.2023.1217449 (PMC10399763; doi:10.3389/fmicb.2023.1217449)
Supplement: Supplementary file 2 [file Data_Sheet_1.docx]

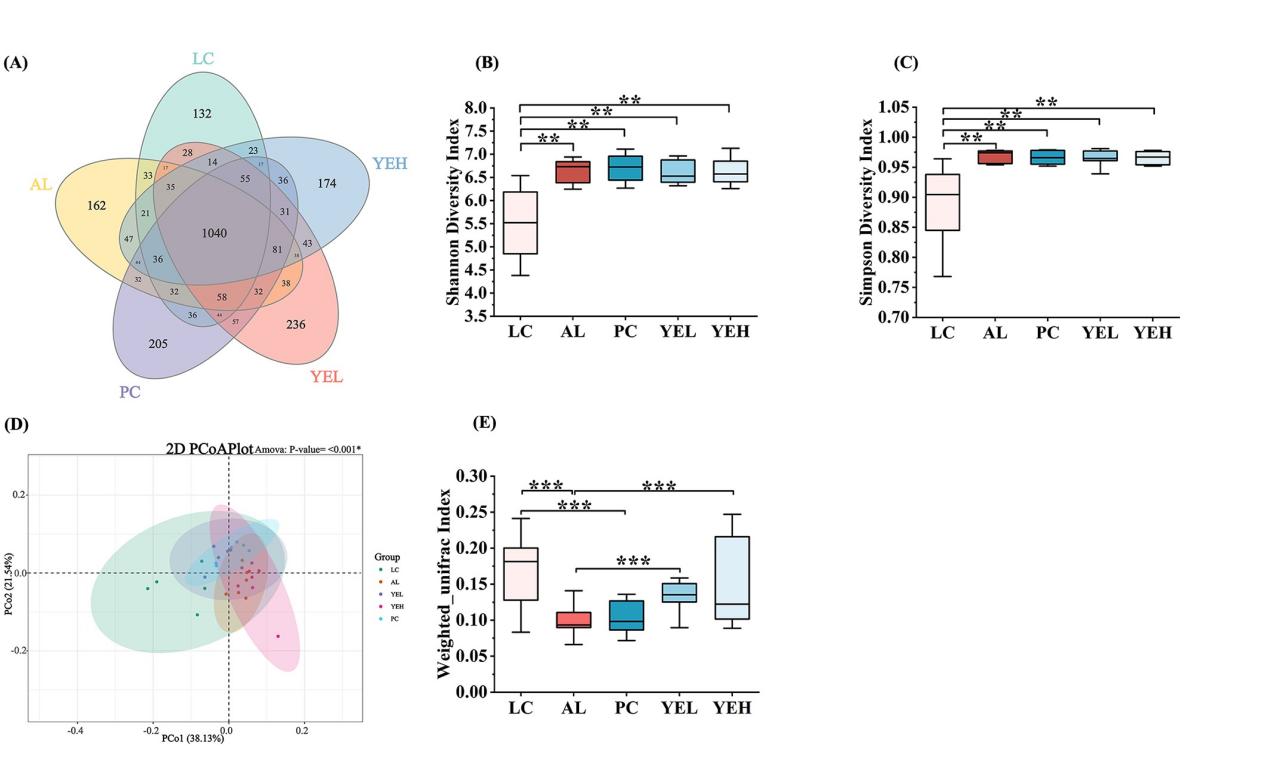


**Supplementary Figure 1.** Yeast extract adjusted overall structure of gut microbiota. (A) The Venn diagram depicted the delivery of OTUs among different groups. (B) Shannon diversity index of gut microbiota. (C) Simpson diversity index of gut microbiota. (D) principal-coordinate analysis (PCoA) plot among different groups based on weighted UniFrac distance. Each point represents each sample. (E) Weighted-unifrac index of gut microbiota. Data are expressed as mean ± SD (n =6). *^*^P* <0.05, ^**^*P* < 0.01, ^***^*P* < 0.001

**Supplementary. Table. 1** Nutrient composition of yeast extract.

| Nutrient | Content（100g） |
| --- | --- |
| Calorie (kcal) | 106 |
| Protein (g) | 2.60 |
| Fat (g) | 1.70 |
| Carbohydrate (g) | 23.90 |
| Dietary Fiber (g) | 7.90 |
| Niacin (mg) | 4.30 |
| Riboflavin (mg) | 0.81 |
| Retinol Equivalent (4.4μg) | 4.40 |
| Carotene (μg) | 0.80 |
| Vitamin B1 (mg) | 6.56 |
| Vitamin E (mg) | 250.75 |
| Ferrum (mg) | 7.10 |
| Kalium（mg） | 448.00 |
| Magnesium (mg) | 7.10 |
| Calcium (mg) | 9.00 |
| Phosphorus (mg) | 409.00 |
| Zinc (mg) | 3.08 |
| Natrium (mg) | 13.60 |
| Manganese (mg) | 0.63 |
| Copper (mg) | 20.12 |
| Selenium (mg) | 2.82 |


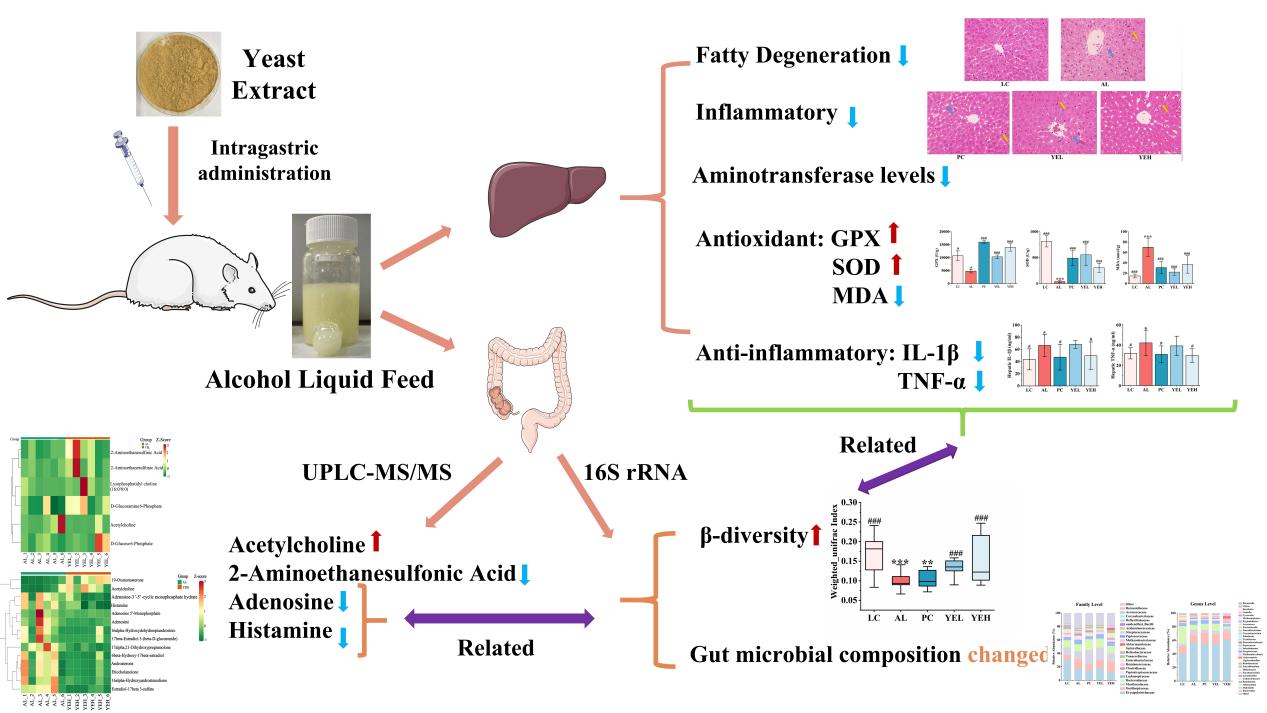


Graphical abstract
